# GRg1 inhibits the TLR4/NF-kB signaling pathway by upregulating miR-216a-5p to reduce growth factors and inflammatory cytokines in DR

**DOI:** 10.1007/s11033-023-08895-3

**Published:** 2023-10-11

**Authors:** Liping Xue, Min Hu, Qin Zhu, Yadi Li, Guanglong Zhou, Xiaofan Zhang, Yuan Zhou, Jieying Zhang, Peng Ding

**Affiliations:** 1 Department of Pediatric Ophthalmology, The Affiliated Hospital of Yunnan University; The Second People’s Hospital of Yunnan; The Affiliated Ophthalmology Hospital of Yunnan University, Kunming, 650021 Yunnan China; 2 Department of Neurosurgery, The First Affiliated Hospital of Kunming Medical University, Kunming, 650032 Yunnan China

**Keywords:** GRg1, miR-216a-5p, TLR4/NF-kB, Inflammatory factor, Growth factor, Angiogenesis

## Abstract

**Background:**

Diabetic retinopathy (DR) is a common diabetic neurodegenerative disease that affects vision in severe cases. Current therapeutic drugs are ineffective for some patients with severe side effects, and ginsenoside-Rg1 (GRg1) has been shown to protect against DR and may serve as a new potential drug for DR. This study aimed to confirm the protective effect of GRg1 against DR and its molecular mechanism.

**Methods:**

Human retinal microvascular endothelial cells (hRMECs) and rats were used to construct DR models in vitro and in vivo. Cell proliferation was detected by BrdU assays, the cell cycle was detected by flow cytometry, and TNF-α, IL-6 and IL-1β levels were detected by ELISA. qRT‒PCR, Western blotting and immunohistochemistry were used to detect the expression of related genes and proteins, and angiogenesis assays were used to assess angiogenesis. RIP and RNA pull down assays were used to determine the relationship between miR-216a-5p and TLR4; retinal structure and changes were observed by HE staining and retinal digestive spread assays.

**Results:**

GRg1 effectively inhibited HG-induced hRMEC proliferation, cell cycle progression and angiogenesis and reduced the levels of intracellular inflammatory cytokines and growth factors. HG downregulated the expression of miR-216a-5p and upregulated the expression of TLR4/NF-kB signaling pathway-related proteins. Importantly, GRg1 inhibited TLR4/NF-kB signaling pathway activation by upregulating miR-216a-5p, thereby inhibiting HG-induced cell proliferation, cell cycle progression, angiogenesis, and the production of inflammatory cytokines and growth factors. In addition, animal experiments confirmed the results of the cell experiments.

**Conclusions:**

GRg1 inhibits TLR4/NF-kB signaling by upregulating miR-216a-5p to reduce growth factors and inflammatory cytokines in DR, providing a potential therapeutic strategy for DR.

## Introduction

Diabetic retinopathy (DR) is a common diabetic neurodegenerative disease accompanied by inflammation and microvascular complications that causes irreversible damage to retinal neurons, glial cells and the microvasculature and seriously threatens the vision of patients. Approximately 33.3% of people with diabetes have some degree of DR, and 1 in 10 progress to vision-threatening levels [1]. There is growing interest in the role of inflammation and angiogenesis in DR-associated structural and molecular alterations, which are involved in the development of diabetic visual impairment [2]. However, DR is an asymptomatic disease, and there is no treatment for the early stages [3]. Advanced DR is treated by vascular endothelial growth factor (VEGF) inhibitors in the clinic, but some patients have poor therapeutic outcomes and serious side effects [4]. Therefore, exploring the molecular mechanisms to effectively improve DR and regulate growth factors and inflammatory factors can provide new strategies for the treatment and prevention of DR.

Human ginsenoside-Rg1 (GRg1) is the active component of ginseng. In recent studies, GRg1 was shown to exert a strong retinal protective effect against the progression of DR and may be used for early DR treatment. Ye Gao et al. [5] found that GRg1 reduced cell apoptosis and prevented diabetic retinopathy in the ganglion cell layer or nuclear layer. DR research by Ying Ying et al. reported that GRg1 could prevent diabetic retinal synaptic neurodegeneration by activating IRS-1/Akt/GSK3β signaling in the early stages [6]. In addition, inflammation and angiogenesis play key roles in the pathogenesis of DR. Inflammation is present in different stages of DR. Diabetes leads to an increase in different stages of DR, and diabetes causes local and systemic increases in many inflammatory molecules involved in the development of DR, such as vascular adhesion molecules, cytokines, chemokines, transcription and growth factors [7–9], through common mediators and signaling pathways associated with inflammation and angiogenesis [10], resulting in an increase in retinal vascular permeability and neovascularization. GRg1 exerts anti-inflammatory effects by inhibiting the increase in proinflammatory cytokines and influencing the activity of inflammatory signaling pathways such as NF-kB [11]. However, little is known about the regulatory effect of GRg1 on inflammatory cytokines and growth factors in DR.

TLR4 is expressed in neurons, microglia, and astrocytes. By activating NF-κB signaling, TLR4 is involved in the release of inflammatory mediators [12]. The TLR4/NF-kB pathway was shown to have an important regulatory role in the inflammatory response [13], cell damage [14], autophagy [15], and neuroprotection [16]. In addition, activating the TLR signaling pathway can stimulate VEGF expression and blood vessel formation in endothelial cells [17]. Studies have shown that inhibiting the activity of the TLR4/NF-κB signaling pathway can inhibit the inflammatory response [18]. Dysregulation of TLR4 signaling plays an important role in the development and progression of various diseases, such as ischemia‒reperfusion injury, atherosclerosis, hypertension, cancer, and neuropsychiatric and neurodegenerative diseases [19]. In addition, the TLR4/NF-kB signaling pathway has been shown to be involved in the regulation of DR [20–22]. However, there has been little research on the specific molecular mechanisms of TLR4/NF-kB in DR. Therefore, it is crucial to explore the molecular regulatory mechanism of TLR4/NF-kB in DR to prevent changes in the neuroretina and maintain visual function.

As small noncoding RNAs, miRNAs can be paired with the 3'UTR sequence of target mRNAs and participate in the regulation of gene expression at the posttranscriptional level. MiRNAs such as miR-23a [23], miR-27b-3p [24], and miR-138-5p [25] play an indispensable role in DR development, which has been proven by an increasing number of studies. In this study, miR-216a-5p was differentially expressed in diabetic retinopathy tissues, and so we hypothesized that the progression of DR might be regulated by miR-216a-5p. In addition, miR-216a-5p has been reported to regulate cell damage, oxidative stress and inflammation. Furthermore, Rui et al. [26] found that miR-216a-5p could regulate inflammatory cytokine production and TLR4 signaling pathway activity by binding to the 3'-UTR of TLR4. There was a targeting relationship between miR-216a-5p and the NF-kB signaling pathway in Yin et al. [27]. In addition, in Ran Yin et al. [28], the regulatory effect of GRg1 on miRNA was observed in Alzheimer’s disease: neuronal apoptosis could be inhibited by GRg1 and AGR by regulating the expression of miR-873-5p. In conclusion, we hypothesize that GRg1 can affect the TLR4/NF-kB signaling pathway by regulating miR-216a-5p, thus affecting the expression of growth factors and inflammatory cytokines in DR and regulating the progression of DR.

## Experimental methods

### Cell treatment and culture

Human retinal microvascular endothelial cells (hRMECs) were purchased (American Type Culture Collection, Inc.) and cultured in Dulbecco’s modified Eagle medium (DMEM, Gibco) containing fetal bovine serum (10%) and streptomycin (1%). The cells were placed at 37 °C with 95% relative humidity under 5% CO_2_. D-glucose was added to the medium at a final concentration of 30 mmol/L to produce high glucose (HG) conditions, and a concentration of 5 mM glucose was the normal glucose condition. After 48 h, 10 μM Rg1 was added and incubated for another 48 h. Lipofectamine™ 2000 (Thermo Fisher Scientific, USA) was used to transfect the miR-216a-5p mimic, miR-216a-5p inhibitor, and NC inhibitor into these cells when they reached approximately 80% confluence.

### STZ-induced DR rat model

Eighty 10-week-old (250–300 g) SPF male rats were prepared, and none of the rats had any eye diseases after examination. Sixty rats were fed a high-fat diet, and 20 control rats were fed a normal diet. The rats were fasted 12 h before drug administration by intraperitoneal injection. Rats that were fed a high-fat diet were injected with STZ (streptozotocin, Sigma, USA) and treated with citrate buffer (0.1 M, pH 4.5, 60 mg/kg). After 3 d, blood samples were collected from the tail vein, and an off-line blood glucose monitoring system was used to measure blood glucose levels. The control rats received the same treatment, and the blood glucose level was also measured. The blood glucose level exceeded 16.7 mmol/L for one week, indicating that the diabetic rat model was successfully established. In the Rg1 treatment group, 0.5 ml of (5 g/ml) Rg1 solution was administered daily, and the control group was given the same dose of saline. After 16 weeks of modeling, the rats were euthanized, and serum and fresh retinal tissue were collected for follow-up experiments.

### BrdU staining

BrdU was incubated with the cells for 30 min. After that, 70% ethanol was used to suspend the cells at 4 °C for 30 min, and the cells were fixed and pelleted. Subsequently, the cells were centrifuged to remove the supernatant, washed once with PBS, and incubated with freshly prepared 2 M HCl for 30 min at 25 °C. The cells were washed twice with PBS and then resuspended in PBS-Tween buffer (containing 0.2% Tween 20 and 0.1% BSA, pH 7.4). Subsequently, the anti-BSA BrdU monoclonal antibody was added to the cell suspension and incubated at 25 °C in the dark for 20 min. Finally, the samples were washed twice with PBS-Tween, and RNAse was incubated with the cell pellet for 15 min at 25 °C. Photographs were taken with a fluorescence microscope.

### Flow cytometry

We used flow cytometry to examine the cell cycle. In brief, the cells were cultured in 6-well plates until the confluence reached 60–70%, and 5 × 10^5^ cells were collected. Then, 70% ethanol was used to fix the cells at 4 °C for 2 h. After the cells were centrifuged for 5 min, precooled PBS was used to precipitate and resuspend the cells, and the supernatant was removed. In the dark, propidium iodide staining solution was added, and the cell pellet was slowly and fully resuspended and incubated at 37 °C for 30 min. Finally, flow cytometry was used at an excitation wavelength of 488 nm to detect red fluorescence.

### Enzyme-linked immunosorbent assay (ELISA)

After the indicated treatments, cell supernatant was collected, and rat serum was collected. ELISA kits (MlBIO, Shanghai, China) were used to detect the tumor necrosis factor-α (TNF-α), interleukin-6 (IL-6), and interleukin-1β (IL-1β) levels using 100 μL of lysate in the ELISA plates for 2 h. Then, corresponding antibodies were added and incubated for 1 h. After the ELISA plates were washed, they were incubated for 20 min with horseradish peroxidase (HRP)-streptavidin. The absorbance values were measured at 450 nm by a microplate spectrophotometer.

### Western blotting

Proteins were extracted with RIPA lysis buffer (Sangon Biotech, Shanghai), and benzoyl fluoride (PMSF) was added. A BCA assay (Sangon Biotech, Shanghai) was used to determine the total protein concentration. Equal amounts of extracted proteins were added to the loading buffer and heated to 95 °C for 10 min, and then the proteins were separated by 10% polyacrylamide gel. The wet transfer method was used to transfer the proteins to PVDF membranes, which were blocked in 5% bovine serum protein for 1 h. Subsequently, primary antibodies (Abcam, UK, 1:1000) against VEGF, FGF, PDGF, TLR4, MYD88, TRAF6, p-NF-KB and NF-KB were added and incubated overnight at 4 °C. Subsequently, the primary antibodies were removed, and the membranes were washed three times with wash buffer for 5 min each. Secondary antibodies (1:1000, ab205718) were added and incubated for 2 h at 4 °C. The control protein was GAPDH. Subsequently, chemiluminescent reagents were added, and the grayscale values of the bands were analyzed using ImageJ software. Three independent experiments were performed.

### Tubule formation experiment

A tubule formation assay was performed on hRMECs. Matrigel was added to 96-well plates and placed in an incubator for 0.5 h. Subsequently, the cells were digested to prepare a cell suspension. Fifty microliters of the cell suspension was added to each well of a 96-well plate, incubated for 12 h, photographed, and analyzed.

### qRT‒PCR

Total RNA was extracted using a Total RNA Extractor (Sangon Biotech). The integrity of 1 μl of RNA was examined by 1% agarose gel electrophoresis, and 1 μl of RNA sample was diluted to measure the OD value; the OD260/OD280 ratio was used to identify total RNA purity. A cDNA synthesis kit (Vazyme, Nanjing, China) was used to reverse transcribe 2 μg of RNA into cDNA, which was diluted 10 times. One microliter of the prepared cDNA was used for qPCR. U6 was used as the reference gene and was analyzed on an ABI7500 real-time PCR system. The qRT‒PCR conditions were as follows: 95 °C for 30 s, 3 s at 95 °C, followed by annealing at 60 °C for 30 s for 40 cycles. All primers (Table 1) used in this study were designed with Premier 5.0. The results were calculated by the 2^−ΔΔCt^ method and were repeated at least 3 times.

**Table 1:** Primer sequences

Genes	Forward primer	Reverse primer
miR-216a-5p	5′-AGGCTGGCCGTGATGAATT-3′	5′-GAGAGCCGTGTATGACTCGCT-3′
U6	5′-CGATACAGAGAAGATTACATGGC-3′	5′-AACGCTTCACGAATTTGCGT-3′

### RNA binding protein immunoprecipitation (RIP)

Anti-TLR4 and anti-IgG antibodies were used to verify the interaction of TLR4 with miR-216a-5p. In brief, 2 × 10^7^ cells were harvested and lysed using an RNA-binding protein immunoprecipitation kit (Millipore, MA, USA). The lysates were incubated with antibodies against TLR4 and IgG for 16 h at 4 °C, and samples were obtained for analysis by RT‒qPCR.

### RNA pull down assay

Biotin-labeled miR-216a-5p-specific probes were synthesized, and 2 × 10^7^ cells were harvested, lysed, and prewashed to remove impurities using an RNA pulldown kit (GenePharma, China). Streptavidin magnetic beads were incubated with the probe for 30 min at 25 °C to obtain a probe bead complex. The cell lysates were then incubated with probe bead complexes at 25 °C for 2 h with rotation, elution buffer was used to wash the beads at 37 °C for 2 h to obtain pull-down samples, and the pull-down products were analyzed by Western blotting.

### HE staining

Paraffin sections of rat retinas were dewaxed, hydrated, and cleaned with distilled water. Then, hematoxylin staining was performed for 2 min, the samples were washed with distilled water, acid water and ammonia water were used for color separation, and the samples were washed with water. Eosin staining was performed for 1 min, the samples were washed with water, dehydrated with gradient alcohol, cleared with xylene, sealed with neutral gum, and observed and photographed under an optical microscope.

### Rat retinal digestive spread staining

After sixteen weeks, the rats were euthanized, and 4% paraformaldehyde was used to fix the eyeballs for 48 h. The sclera was cut from the posterior ciliary body near the serrate margin, and the lens and vitreous body were removed. The posterior segment of the eye was divided into three equal parts, and the retina was gently rinsed in water for 3 h. Then, 3% trypsin digestion solution was added and incubated at 37 °C for 3 h. The retina was gently placed in distilled water and agitated so that the residual retinal nerve components and internal limiting membrane were completely removed, leaving a layer of the transparent retinal vascular network. The retina was moved to the slide as far as possible and stained with isolectin B4 (IB4). The samples were observed and photographed with a fluorescence microscope.

### Immunohistochemistry

After dewaxing and hydration, antigenic repair was carried out with sodium citrate antigenic repair solution, and then the sections were treated with hydrogen peroxide for 10 min. Nonspecific binding sites were blocked with serum from the same source as the secondary antibody, and the diluted primary antibody was added dropwise and incubated overnight at 4 °C. The slices were washed with PBS and incubated with secondary antibodies at 37 °C for 2 h. DAB was used for color development, followed by staining with Mayer hematoxylin, washing with water, differentiation with hydrochloric acid and alcohol, and dehydration neutral gum was added to seal the slides, and images were taken.

### Statistical analysis

All experiments were repeated at least 3 times. GraphPad Prism 8.0 was used for all statistical analyses. The mean value and standard deviation (SD) are shown. Student’s t test and one-way ANOVA were used to analyze the differences between groups, and a *P* < 0.05 was considered statistically significant.

## Results

### GRg1 reduces inflammatory cytokines and growth factors in retinal microvascular endothelial cells

**Fig. 1:**
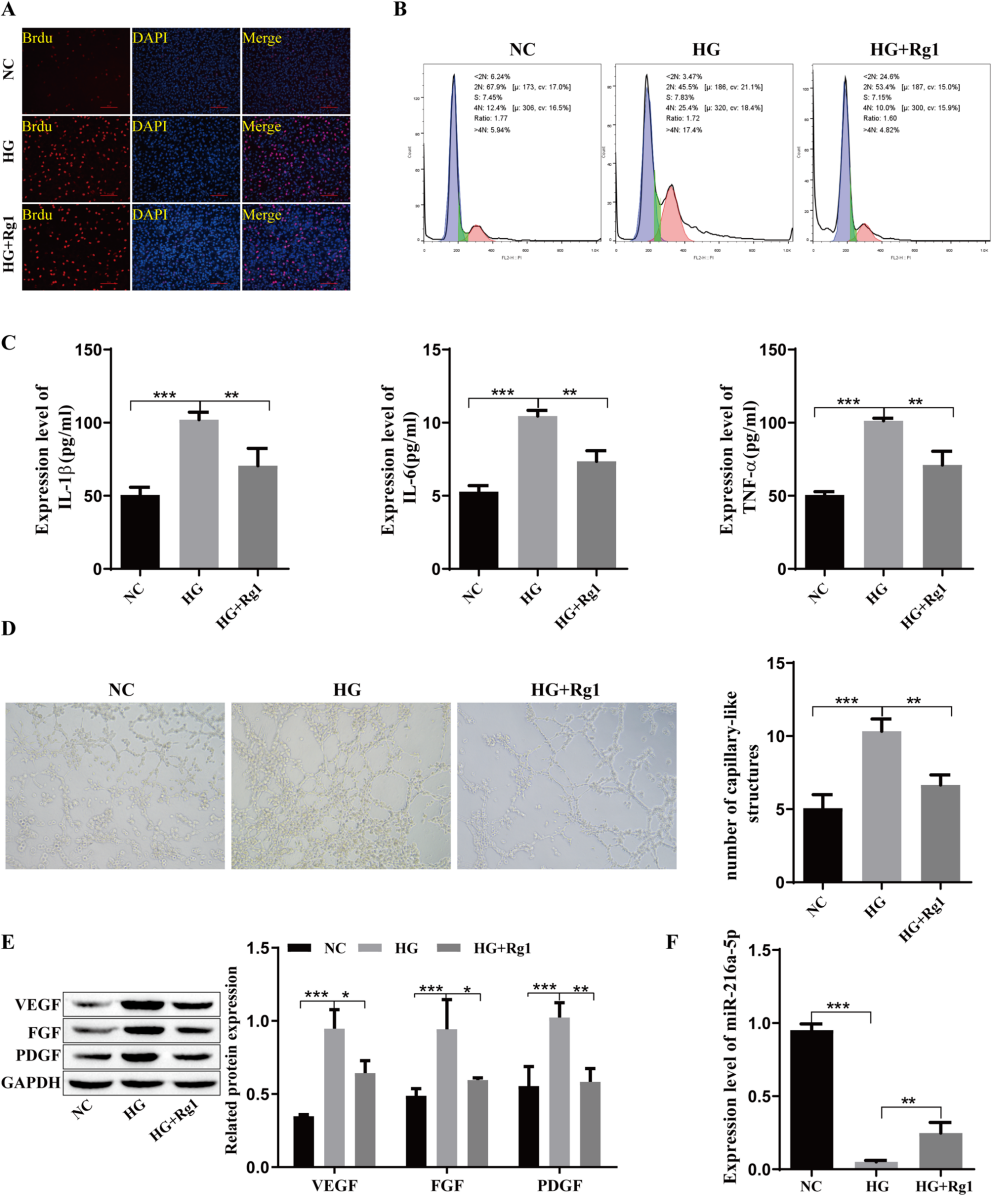
Effect of GRg1 on inflammatory cytokines and growth factors in retinal microvascular endothelial cells. **A**: BrdU staining to detect cell proliferation. **B**: Flow cytometry to detect the cell cycle. **C**: ELISA to detect the levels of inflammatory cytokines TNF-α, IL-6 and IL-1β. **D**: Angiogenesis was detected by tubule formation experiments. **E**: Western blot analysis of the growth factors VEGF, FGF and PDGF. F: qRT‒PCR analysis of miR-216a-5p. *P<0.05, **P<0.01, ***P<0.001

To investigate the effect of GRg1 on DR, a DR cell model was established, and the cells were treated with Rg1. As shown in Fig. 1A, BrdU staining was used to detect cell proliferation, and the results showed that HG could promote the proliferation of cells. As expected, after adding Rg1, cell proliferation was reduced. Flow cytometry showed that HG significantly inhibited G0/G1 stagnation and promoted cell cycle progression. The addition of Rg1 weakened the effect of HG (Fig. 1B). The ELISA results showed that the levels of TNF-α, IL-6 and IL-1β were high in the HG group, and they were reduced by Rg1 (Fig. 1C). The angiogenesis experiment showed that Rg1 could effectively inhibit angiogenesis induced by HG (Fig. 1D). The growth factors VEGF, FGF, and PDGF were highly expressed in the HG group, as shown by Western blotting, whereas the expression levels of these factors were significantly reduced by Rg1 (Fig. 1E). We also examined the level of miR-216a-5p by qRT‒PCR. The level of miR-216a-5p was decreased by HG conditions and increased after the addition of Rg1 (Fig. 1F). In conclusion, HG promoted the proliferation of hRMECs, promoted cell cycle progression, enhanced the levels of inflammatory cytokines and growth factors, intensified angiogenesis, and inhibited the production of miR-216a-5p, and the effects of HG were weakened by the addition of Rg1.

### MiR-216a-5p overexpression can reduce the levels of inflammatory cytokines and growth factors in retinal microvascular endothelial cells

**Fig. 2:**
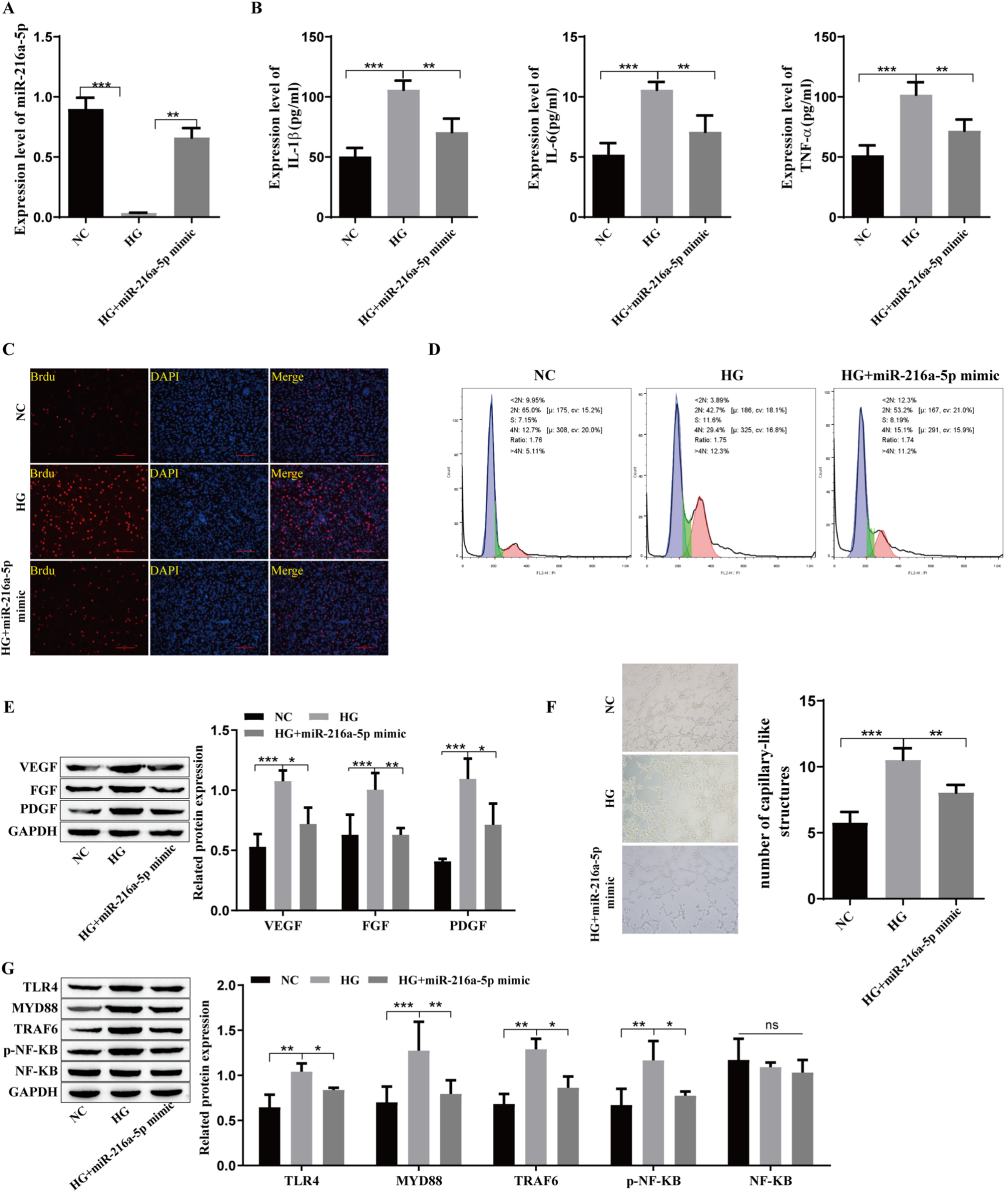
Influence of miR-216a-5p overexpression on inflammatory cytokines and growth factors in retinal microvascular endothelial cells. **A**: qRT‒PCR analysis of the level of miR-216a-5p. **B**: The expression of the inflammatory cytokines TNF-α, IL-6 and IL-1β was detected by ELISA. **C**: BrdU staining to detect cell proliferation. **D**: Flow cytometry to detect the cell cycle. **E**: Western blot analysis of the expression levels of the growth factors VEGF, FGF and PDGF. **F**: Angiogenesis was detected by tubule formation experiments. **G**: Western blot analysis of the level of TLR4/NF-kB-related proteins such as TLR4, MYD88, TRAF6, p-NF-kB and NF-kB. *P<0.05, **P<0.01, ***P<0.001

After determining that GRg1 could upregulate miR-216a-5p, a miR-216a-5p mimic was transfected into hRMECs to investigate the effects of miR-216a-5p overexpression on inflammatory factors and growth factors in hRMECs. The level of miR-216a-5p was significantly higher than that in the HG group after transfection with the miR-216a-5p mimic (Fig. 2A). The ELISA results showed that the levels of TNF-α, IL-6 and IL-1β were high in the HG group, and they were reduced after the upregulation of miR-216a-5p (Fig. 2B). HG promoted cell proliferation, and cell proliferation was significantly inhibited after transfection of the miR-216a-5p mimic (Fig. 2C). Flow cytometry showed that miR-216a-5p overexpression induced G0/G1 stagnation and inhibited cell cycle progression (Fig. 2D). The growth factors VEGF, FGF, and PDGF were highly expressed in the HG group, as shown by Western blotting, and the expression levels of these factors were significantly reduced after the upregulation of miR-216a-5p (Fig. 2E). Angiogenesis experiments showed that miR-216a-5p overexpression weakened the effect of HG and inhibited angiogenesis (Fig. 2F). TLR4/NF-kB signaling pathway-related proteins were examined, and the results showed that TLR4, MYD88, TRAF6, and p-NF-kB were highly expressed in the HG group, and their expression levels were decreased after miR-216a-5 was upregulated, but there was no significant change in NF-kB. (Fig. 2G). These findings suggest that the high level of miR-216a-5p reduced the levels of inflammatory and growth factors in retinal microvascular endothelial cells.

### Inhibition of the TLR4/NF-kB signaling pathway reduces growth factors and inflammatory cytokines in retinal microvascular endothelial cells

**Fig. 3:**
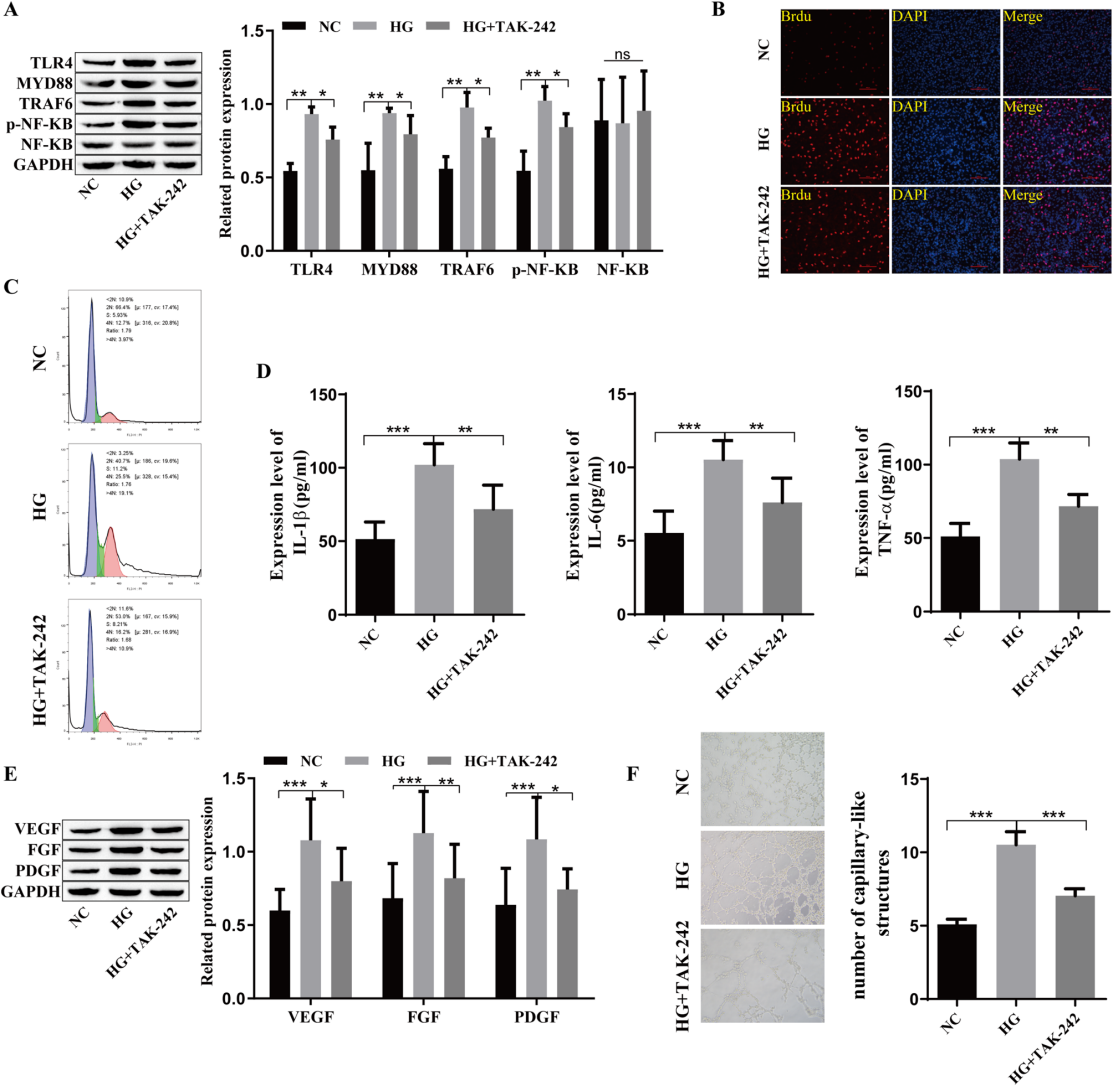
Inhibitory effects of the TLR4/NF-kB signaling pathway on growth factors and inflammatory cytokines in retinal microvascular endothelial cells. **A**: Western blotting was used to detect the levels of TLR4/NF-kB-related proteins. **B**: BrdU staining was used to detect cell proliferation. **C**: Flow cytometry was used to detect the cell cycle. **D**: ELISA was used to detect the levels of TNF-α, IL-6 and IL-1β. **E**: Western blotting was used to detect the levels of VEGF, FGF and PDGF. F: The tubule formation assay was used to detect neovascularization. *P<0.05, **P<0.01, ***P<0.001

To verify the influence of the TLR4/NF-kB signaling pathway on inflammatory factors and growth factors in diabetic retinopathy, we used the TLR4/NF-kB signaling inhibitor TAK-242. The levels of TLR4/NF-kB-related proteins were detected by Western blotting, and compared with that in the HG group, TAK-242 significantly inhibited the expression of TLR4, MYD88, TRAF6 and p-NF-kB (Fig. 3A). BrdU staining showed a significant increase in cell proliferation in the HG group, and the addition of TAK-242 significantly decreased cell proliferation (Fig. 3B). Flow cytometry showed that compared with that in the HG group, TAK-242 induced G0/G1 stagnation and inhibited cell cycle progression (Fig. 3C), and TAK-242 decreased the levels of IL-1β TNF-α and IL-6 (Fig. 3D). Western blot analysis showed that the levels of the growth factors VEGF, FGF, and PDGF were increased in the HG group, and the levels were significantly decreased after the addition of TAK-242 (Fig. 3E). Angiogenesis experiments showed that TAK-242 inhibited angiogenesis compared with that in the HG group (Fig. 3F). Inhibiting the TLR4/NF-kB signaling pathway could reduce the levels of inflammatory and growth factors in retinal microvascular endothelial cells.

### Interaction between miR-216a-5p and TLR4

**Fig. 4:**
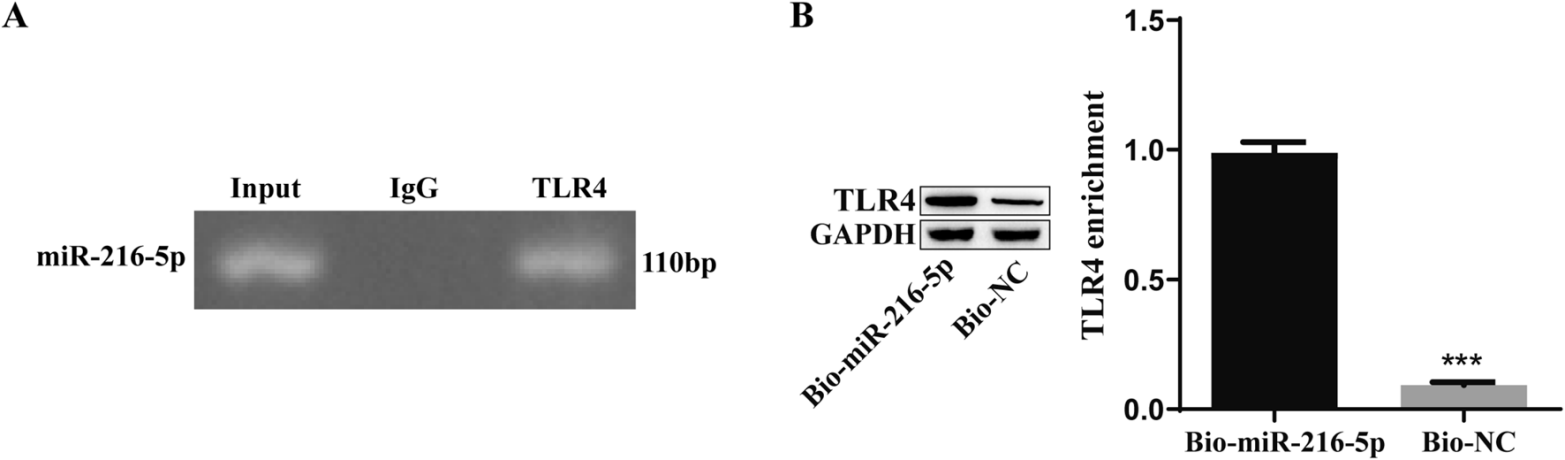
Verification of the interaction between miR-216a-5p and TLR4. A: RIP verified that TLR4 could effectively enrich miR-216a-5p. B: RNA pull-down assay showing that TLR4 was specifically pulled down by the miR-216a-5p probe. ***P < 0.001 compared to the NC group

RIP was used to determine whether TLR4 interacts with miR-216a-5p, and the results showed that TLR4 could efficiently enrich miR-216a-5p (Fig. 4A). In addition, we used a biotin-labeled miR-216a-5p-specific probe, performed an RNA pulldown assay, analyzed the pulldown products by Western blotting, and confirmed that TLR4 was specifically pulled down by the miR-216a-5p probe (Fig. 4B). The interaction between miR-216a-5p and TLR4 was verified by these results.

### GRg1 reduces inflammatory cytokines and growth factors in retinal microvascular endothelial cells by upregulating miR-216a-5p

**Fig. 5:**
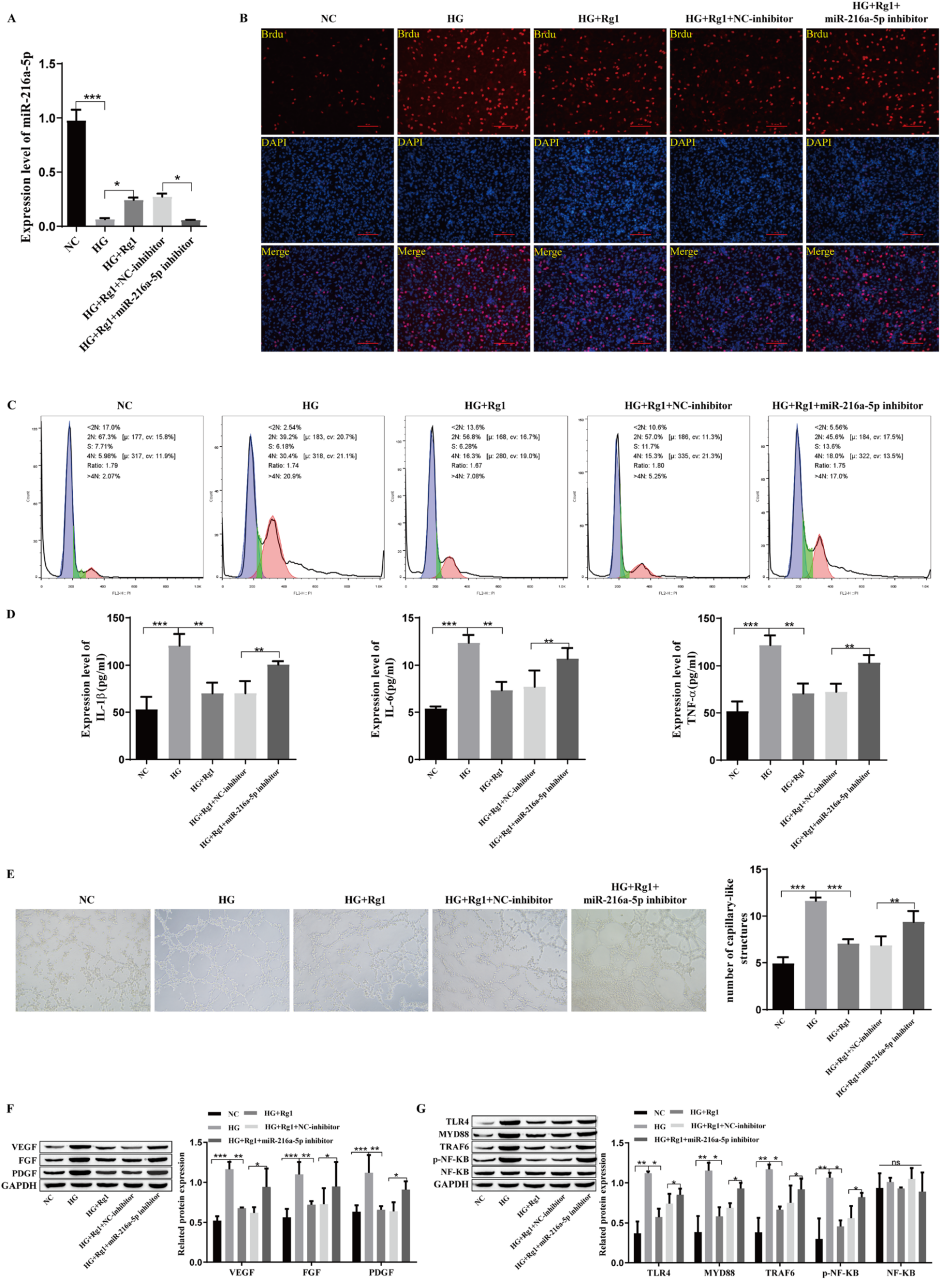
Effect of GRg1 on inflammatory cytokines and growth factors in retinal microvascular endothelial cells through the upregulation of miR-216a-5p. **A**: The level of miR-216a-5p was detected by qRT‒PCR. **B**: Cell proliferation was detected by BrdU staining. **C**: The cell cycle was detected by flow cytometry. **D**: The levels of the inflammatory cytokines TNF-α, IL-6 and IL-1β were detected by ELISA. **E**: Angiogenesis was detected by tubule formation experiments. **F**: The levels of the growth factors VEGF, FGF and PDGF were detected by Western blotting. **G**: The levels of TLR4/NF-kB-related proteins were detected by Western blotting. *P<0.05, **P<0.01, ***P<0.001

A miR-216a-5p inhibitor was transfected into Rg1-treated hRMECs to confirm the regulator relationship between miR-216a-5p and GRg1, and the results were measured by qRT‒PCR. Compared to that in the HG + Rg1 group, miR-216a-5p expression was significantly reduced after transfection of the miR-216a-5p inhibitor (Fig. 5A). The cell proliferation results showed that HG promoted cell proliferation, cell proliferation was reduced by Rg1, and cell proliferation was significantly enhanced by miR-216a-5p inhibitor transfection (Fig. 5B). Cell cycle analysis showed that compared with that in the HG group, Rg1 could significantly induce G0/G1 stagnation and inhibit cell cycle progression, while transfection of the miR-216a-5p inhibitor weakened the effect of Rg1 and further promoted cell cycle progression. (Fig. 5C). ELISA results showed that TNF-α, IL-6 and IL-1β were highly expressed in the HG group, and Rg1 could reduce the level of inflammatory factors, which was increased after transfection of the miR-216a-5p inhibitor (Fig. 5D). Angiogenesis experiments showed that HG promoted angiogenesis, and Rg1 weakened the effect of HG, but after transfection of the miR-216a-5p inhibitor, the effect of Rg1 was weakened, and angiogenesis was promoted. (Fig. 5E). The growth factors VEGF, FGF, and PDGF were highly expressed in the HG group, as shown by Western blotting, and these factors were decreased after the addition of Rg1, while the expression of these factors was increased after transfection of the miR-216a-5p inhibitor (Fig. 5F). Western blot analysis was performed to detect the expression of TLR4/NF-kB-related proteins. The results showed that compared with that in the HG group, the expression of TLR4, MYD88, TRAF6 and p-NF-kB was significantly inhibited by Rg1, and the effect of Rg1 was weakened after transfection of the miR-216a-5p inhibitor. The miR-216a-5p inhibitor promoted the expression of these proteins and had no significant effect on the expression of NF-kB. (Fig. 5G). These results suggest that GRg1 can inhibit the levels of inflammatory cytokines and growth factors in diabetic retinopathy by promoting the expression of miR-216a-5p.

### GRg1 promotes the expression of miR-216a-5p, inhibits the TLR4/NF-kB signaling pathway, and reduces the expression of growth factors and inflammatory cytokines in retinal microvascular endothelial cells

**Fig. 6:**
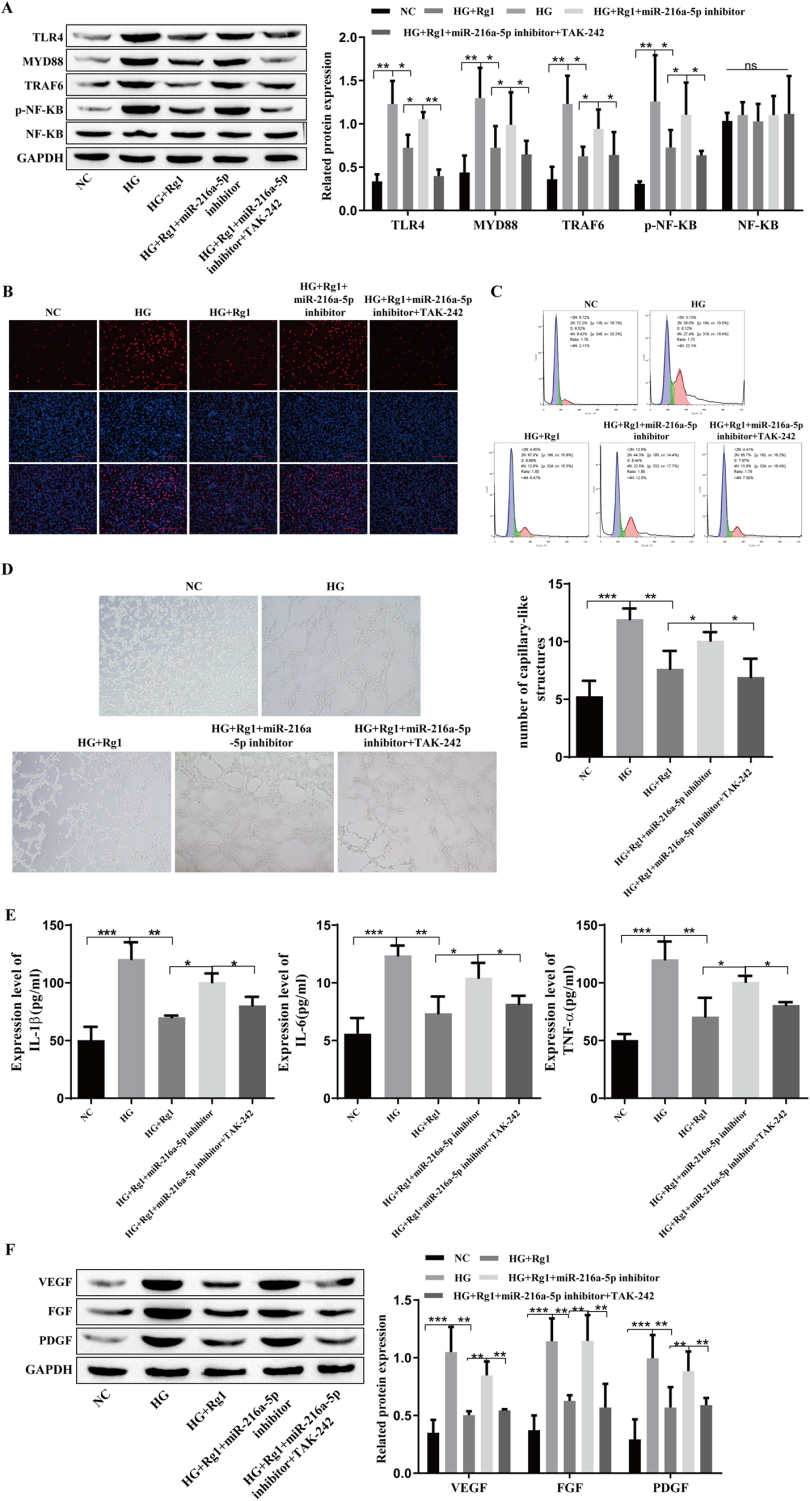
GRg1 promotes the expression of miR-216a-5p and inhibits the TLR4/NF-kB signaling pathway to affect growth factors and inflammatory cytokines in retinal microvascular endothelial cells. **A**: The levels of TLR4/NF-kB-related proteins were detected by Western blotting. **B**: Cell proliferation was detected by BrdU staining. **C**: The cell cycle was detected by flow cytometry. **D**: Angiogenesis was detected by tubule formation experiments. **E**: The levels of TNF-α, IL-6 and IL-1β were detected by ELISA. **F**: The levels of VEGF, FGF and PDGF by were detected Western blotting. *P<0.05, **P<0.01, ***P<0.001

To further verify the regulatory role of GRg1, miR-216a-5p and TLR4/NF-kB signaling pathways, we added the TLR4/NF-kB pathway inhibitor TAK-242 to hRMECs that were transfected with miR-216a-5p inhibitor and treated with Rg1. The levels of TLR4, MYD88, TRAF6, and p-NF-kB were increased in the HG group, as shown by Western blotting, were decreased by Rg1, and were increased after transfection of the miR-216a-5p inhibitor. The addition of TAK-242 decreased the expression level, and there was no significant effect on the expression of NF-kB (Fig. 6A). BrdU staining showed that HG promoted cell proliferation, and cell proliferation was reduced after the addition of Rg1. After transfection of the miR-216a-5p inhibitor, cell proliferation was enhanced, and cell proliferation was significantly inhibited by TAK-242 (Fig. 6B). Compared with that in the HG group, Rg1 induced G0/G1 stagnation and inhibited cell cycle progression. The effect of Rg1 was weakened after transfection of the miR-216a-5p inhibitor, and G0/G1 stagnation was induced after the addition of TAK-242, thus inhibiting cell cycle progression (Fig. 6C). HG-induced vessel formation was detected by angiogenesis experiments, and Rg1 weakened the effect of HG, promoting angiogenesis after transfection of the miR-216a-5p inhibitor, and the addition of TAK-242 inhibited angiogenesis (Fig. 6D). The ELISA results showed that the expression of inflammatory cytokines in the HG group was high, the expression was reduced after the addition of Rg1, and the level was increased after transfection of the miR-216 a-5p inhibitor, but it was reduced by TAK-242 (Fig. 6E). The Western blot results showed that the levels of VEGF, FGF and PDGF were decreased after the addition of Rg1, and the levels were increased after transfection of the miR-216a-5p inhibitor, and the levels were significantly decreased by TAK-242 (Fig. 6F). In summary, GRg1 promotes miR-216a-5p expression, inhibits TLR4/NF-kB signaling, and reduces the expression of growth factors and inflammatory cytokines in retinal microvascular endothelial cells.

### Animal experiments verify that GRg1 inhibits the TLR4/NF-kB signaling pathway by upregulating miR-216a-5p to reduce growth factors and inflammatory cytokines in DR

**Fig. 7:**
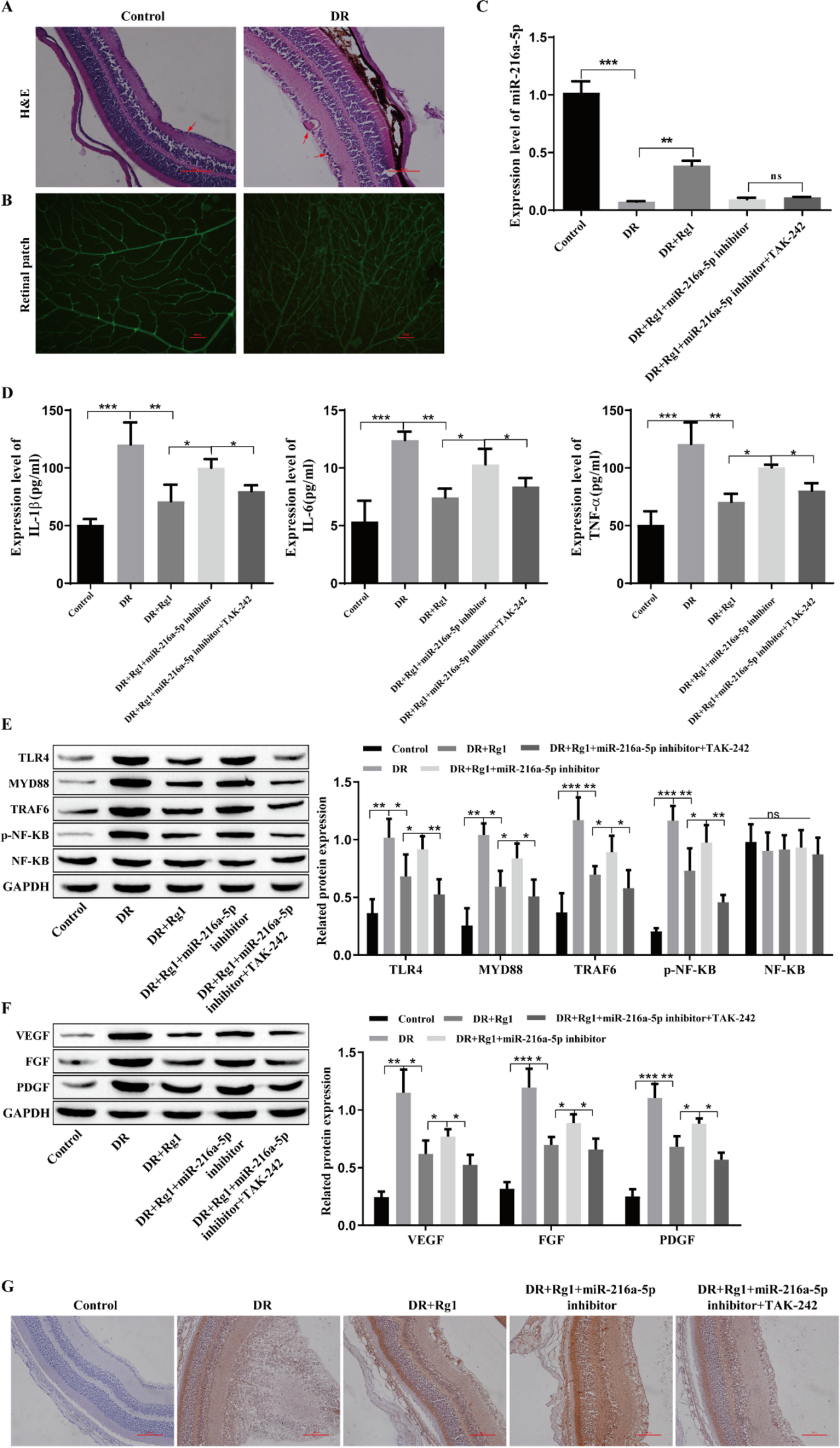
Animal experimental verification of GRg1-mediated inhibition of the TLR4/NF-kB signaling pathway by upregulating miR-216a-5p to reduce growth factors and inflammatory cytokines in DR. **A**: Retinal structure was observed by HE staining. B: Rat retinal digestion and preparation staining. **C**: Detection of the level of miR-216a-5p by qRT‒PCR. **D**: Detection of the levels of TNF-α, IL-6 and IL-1β by ELISA. **E**: Detection of the levels of TLR4, MYD88, TRAF6, p-NF-kB and NF-kB by Western blotting. **F**: Detection of the levels of VEGF, FGF and PDGF by Western blotting. **G**: Detection of the level of VEGF by immunohistochemistry. *P<0.05, **P<0.01, ***P<0.001

HE staining showed that the normal rat retina showed clear structure, orderly distribution and normal shape, and there were fewer vascular endothelial cells near the inner boundary mode of the retina. The retinal surface of DR rats showed edema, increased vascular endothelial cells, and partial vascular dilation (Fig. 7A). The rat retinal digestive patch was stained. The results showed that the number of new blood vessels in the retinas of DR rats was significantly higher than that of normal rats, and a large area of neovascularization sprouts formed between the avascular area and the vascular area of the retina (Fig. 7B). These results indicate that the rat model of DR was successfully established.

The miR-216a-5p inhibitor and TAK-242 were injected into the vitreous cavities of DR rats, and after treatment with GRg1, miR-216a-5p was significantly upregulated in the GRg1-treated groups (Fig. 7C). The ELISA results showed that compared with those in the DR group, the expression levels of TNF-α, IL-6 and IL-1β were decreased after Rg1 treatment, while the effect of Rg1 was weakened after the transfection of the miR-216a-5p inhibitor, and the expression of inflammatory cytokines was inhibited by TAK-242 (Fig. 7D). Western blot analysis showed that the levels of TLR4, MYD88, TRAF6, and p-NF-kB were increased in the DR group. After treatment with Rg1, the expression of these proteins was decreased, and transfection of the miR-216a-5p inhibitor weakened the effect of Rg1, while the addition of TAK-242 reversed the effect of the miR-216a-5p inhibitor, inhibited the expression of these proteins, and had no significant effect on the expression of NF-kB (Fig. 7E). The western blot results showed that the growth factors VEGF, FGF and PDGF were highly expressed in the DR group, and the expression levels of these proteins were decreased by Rg1; the effect of Rg1 was weakened after transfection of the miR-216a-5p inhibitor, while the expression levels of these proteins were significantly decreased after the addition of TAK-242 (Fig. 7F). The level of VEGF was detected by immunohistochemistry, the positive product of VEGF was brown‒yellow granules, and the results were consistent with the Western blot results (Fig. 7G). These results prove that GRg1 inhibits the TLR4/NF-kB signaling pathway by upregulating miR-216a-5p to reduce growth factors and inflammatory factors in DR.

## Discussion

Diabetic retinopathy is becoming one of the main causes of vision loss in the global population [29]. Inflammation and angiogenesis have crucial effects on the progression of DR, and inflammatory and growth factors lead to further pathological processes that ultimately result in vascular permeability (diabetic macular edema) and/or pathologic angiogenesis (proliferative diabetic retinopathy) [30, 31]. In this study, GRg1 was shown to have anti-inflammatory and antitumor effects as the active component of human saponins [32]. GRg1 has been reported to improve lung adenocarcinoma [33], neuroblastoma [34], dermatitis psoriasiform [35], and other diseases. A recent report suggested that GRg1 could prevent early diabetic retinopathy [5]. However, the specific effect and mechanism of GRg1 on diabetic retinopathy remain unclear. We found that GRg1 could inhibit HG-induced hRMEC proliferation and cell cycle progression, reduce the expression of inflammatory cytokines and growth factors, inhibit angiogenesis, and thus play a protective role in DR.

MiRNAs can regulate a variety of biological processes in diabetic retinopathy, especially inflammation, oxidative stress and neurodegeneration [36]. Multiple miRNAs are highly expressed in the retina and are biomarkers or therapeutic targets for retinal diseases [37]. MiR-18b has been reported to exert anti-inflammatory and antiapoptotic effects in DR [38]. MiR-93-5p affects vascular permeability and reduces inflammation and oxidative stress in the retina by targeting Sirt1, ultimately improving retinal pathology and mitigating DR progression [39]. The levels of Bcl-2 and SIRT1 could be upregulated by miR-204 to inhibit inflammation and apoptosis in diabetic retinopathy rats [40]. MiR-216a-5p has a key role in other diseases. For example, targeting TCTN1 with miR-216a-5p could inhibit the proliferation of esophageal squamous cell carcinoma cells and induce apoptosis [41]. As a tumor suppressor, miR-216a-5p targets PAK2 in breast cancer to regulate cell proliferation and metastasis [42]. In this study, it was found that the level of miR-216a-5p in cells was reduced by HG induction. Overexpression of miR-216a-5p inhibited cell proliferation and cell cycle progression, decreased the levels of inflammatory cytokines, growth factors and angiogenesis, and had certain effects on the expression of proteins related to the TLR4/NF-κB signaling pathway. This finding suggests that miR-216a-5p plays a key role in DR. In addition, a study reported that by promoting miR-873-5p expression in Alzheimer’s disease, GRg1 could reduce neuronal apoptosis [42]. These results suggested that GRg1 may play a role in disease by regulating changes in miRNA expression. It is unknown whether GRg1 regulates miR-216a-5p to play a corresponding protective role in diabetes. Our study showed that the expression of miR-216a-5p in cells was increased after GRg1 treatment. Downregulating miR-216a-5p weakens the effect of GRg1 and promotes HG-induced pathological phenomena (cell proliferation, cycle progression, angiogenesis, inflammation, and growth factor production). Thus, we confirmed the regulatory relationship between GRg1 and miR-216a-5p, which have important roles in diabetic retinal disease.

The TLR4/NF-κB signaling pathway mediates the progression of various diseases by regulating inflammation, and suppressing TLR4/NF-κB pathway activation attenuates apoptosis, inflammation, and oxidative stress in HUVECs [43]. Consistent with earlier studies, we inhibited the TLR4/NF-κB signaling pathway with TAK-242 and found that the pathological features of cells and DR rats were reduced. These findings suggested that inhibiting the TLR4/NF-κB signaling pathway can alleviate diabetic retinopathy. Moreover, miR-216a-5p was involved in the regulation of the TLR4/NF-κB signaling pathway, and miR-216a-5p regulates the TLR4/NF-κB/PI3K/AKT signaling cascade and mediates M1/M2 polarization of microglia to repair traumatic spinal cord injury [44]. In this study, we confirmed the interaction between miR-216a-5p and TLR4. MiR-216a-5p can inhibit the expression of the TLR4/NF-kB signaling pathway-related proteins TLR4, MYD88, TRAF6, and p-NF-kB and then inhibit the expression of inflammatory cytokines and growth factors. In conclusion, miR-216a-5p plays a protective role in the occurrence of DR by inhibiting the activation of the TLR4/NF-kB signaling pathway.

In conclusion, this study investigated the molecular mechanism by which GRg1 alleviates DR. We demonstrated that GRg1 inhibited the TLR4/NF-kB signaling pathway by upregulating miR-216a-5p, thereby inhibiting cell proliferation and cell cycle progression, reducing growth factors, inflammatory cytokines and angiogenesis in DR, and preventing retinal damage. These findings provide a theoretical basis for the potential function of GRg1 as a novel therapeutic agent for DR. In a future study, we will explore the effects of different doses of GRg1 on DR through the molecular axis of miR-216a-5p/TLR4/NF-kB in animal experiments.

## Data Availability

The datasets used and/or analyzed during the current study are available from the corresponding author upon reasonable request.
